# School- and Individual-level Predictors of Weight Status Misperception among Korean Adolescents: A National Online Survey

**DOI:** 10.1371/journal.pone.0154826

**Published:** 2016-05-04

**Authors:** Yongjoo Kim, Ichiro Kawachi

**Affiliations:** Department of Social and Behavioral Sciences, Harvard School of Public Health, Boston, Massachusetts, United States of America; Newcastle University, UNITED KINGDOM

## Abstract

**Background:**

Growing body of literature has reported that weight status estimation pattern, including accurate-, under-, and overestimation, was associated with weight related behaviors and weight change among adolescents and young adults. However, there have been a few studies investigating the potential role of school contexts in shaping adolescents’ weight status estimation pattern among Korea adolescents.

**Objective:**

The aim of the present study was to investigate the association between weight status misperception patterns and factors at individual-, family-, and school-level, simultaneously, and whether there was significant between schools variation in the distribution of each weight status misperception pattern, underestimation and overestimation respectively, among Korean adolescents aged 12–18 years.

**Method:**

Data from the Eighth Korea Youth Risk Behavior Web-based Survey (KYRBS), 2012, a nationally representative online survey of 72,228 students (boys = 37,229, girls = 34,999) from a total of 797 middle and high schools were used. Sex stratified multilevel random intercept multinomial logistic models where adolescents (level 1) were nested within schools (level 2) were performed.

**Results:**

At the school level, attending a school with higher average BMI (kg/m^2^) was positively associated with weight status underestimation, and inversely associated with weight status overestimation among boys and girls. Single-sex schooling was positively associated with weight status underestimation among girls. At the family level, higher household income (high/middle versus low) was inversely associated with both weight status under- and overestimation among boys and girls. Higher maternal education (equal to or more than college graduate versus equal to or less than high school graduate) was positively associated with weight status overestimation among boys, and living with both parents (compared to not living with both parents) was inversely associated with weight status underestimation among girls. At the individual level, high academic achievement (compared to low) was positively associated with weight status underestimation among boys and girls.

**Conclusions:**

While further research with prospective designs and objectively measured anthropometric information is needed, school environmental factors such as sex composition and school average BMI, as well as, family contexts such as socioeconomic status need to be considered when developing and implementing obesity prevention programs.

## Introduction

Despite an increase in obesity in South Korea, there is also a substantial problem of weight status overestimation, especially among young women and girls [1–4]. Contrary to a global trend of increasing obesity among adults and adolescents in most developed and developing countries [5–7], average BMI and prevalence of overweight/obesity has been declining among young women during the past decades in South Korea [8, 9]. This is an unique pattern shared by Japan and Taiwan [10, 11].

Weight status misperception (WSM) refers to discrepancy between perceived and actual weight status of an individual, including overestimation, perceived weight status is heavier than actual, and underestimation, perceived weight status is lighter than actual [12]. In terms of weight status overestimation (WSOE), previous studies have reported that WSOE among children and adolescents can lead to negative health consequences such as unhealthy weight control behaviors, depressive symptoms, eating disorders, and suicidal ideation and behaviors [13–16]. At the same time, studies have reported that overweight/obese adolescents with weight status underestimation (WSUE) were less likely to engage in healthy weight control behaviors including regular physical activity and restricting calorie intake [17–21]. However, recent longitudinal studies have reported that weight status overestimation (WSOE) was associated with an increased risk of overweight, while weight status underestimation (WSUE) was associated with a decreased risk of overweight/obesity among US adolescents [12, 22]. In addition, sex difference in the pattern of weight misperception has been reported. Studied reported that women were more likely to overestimate their body weight status while men tended to underestimate their body weight, consistent across Europe, USA, the Mediterranean, Pacific Asia, and South America [23]. Across racial groups, white American girls were more likely to engage in weight control behaviors due to the distorted body image and concerns about being overweight, compared to African American girls and White boys [24, 25].

Among Korean adolescents, the prevalence of weight status misperception was reported as 49.3% (51.1% among boys; 47.5% among girls). Of these, boys were more likely to underestimate (65.0%) while girls were more likely to overestimate (62.2%) [2]. Weight status underestimation (WSUE) was linked to higher probability of having engaged in unhealthy diet, including fast food and unhealthy snacks consumption among both girls and boys, and depressive symptoms among boys, while the association between WSUE and depressive symptoms was not reported to be significant among girls [4, 26]. Weight status overestimation (WSOE) was linked to greater screen time, and higher probability of perceived stress and depressive symptoms among girls, and suicidal ideation among boys and girls [4, 15, 26].

Prior studies reported that individual characteristics including age, sex, race/ethnicity, BMI, and academic performance as well as family contexts including household income and parental education were associated with weight status misperception[4, 27–33]. However, there have been only a few studies investigating potential role of school contexts in shaping weight status estimation. As school environment is an important social context for children and adolescents, which can shape norms regarding health-related behaviors, prior studies have reported the influences of school environments on adolescents’ health and health-related behaviors such as BMI, obesity, depressive symptoms, disordered eating behaviors and smoking [34–42]. In terms of the association between school context and weight status misperception, a prior study presented that higher school average BMI as well as parent BMI were inversely associated with WSUE among Canadian adolescents [28]. However, to our knowledge, there have been little studies investigating the impact of school contexts, simultaneously with family- and individual-level factors, on adolescents’ weight status misperception patterns among Korean adolescents. Therefore, building upon prior studies, in the present study, we aimed to investigate risk and protective factors for each weight status misperception pattern (WSMP) at individual-, family-, and school-level, simultaneously, and whether there was significant between schools variation in the distribution of each weight status misperception pattern (WSMP).

## Methods

### Study Population and Source of Data

We used data from the Eighth Korea Youth Risk Behavior Web-based Survey (KYRBS), administered in June 2012 by the Korean Ministry of Education, Science and Technology, Ministry of Health and Welfare, and Korea Centers for Disease Control and Prevention (KCDC). The KYRBS is a nationally representative school-based online survey, using a stratified multistage cluster-sampling design. The anonymous self-administered survey included 129 questions divided into fifteen sections inquiring about health-related behaviors, mental and physical health [43]. In the 8^th^ KYRBS, a total of 76,980 students from 400 middle and 400 high schools were randomly selected, and 74,186 students (96.4% response rate) from 797 schools (99.6% response rate) responded the survey. The detailed information regarding KYRBS has been reported by the KCDC [43, 44]. In the current study, we excluded 1,958 students due to missing responses (1,957 for weight and height, and 1 for maternal education), leaving an analytical sample of 72,228 students (female = 34,999 and male = 37,229) from a total of 797 middle and high schools. The present study was exempted from IRB review by the Office of Human Research Administration, Harvard School of Public Health as the study used a de-identified publicly available dataset, the Eighth Korea Youth Risk Behavior Web-based Survey, which can be obtained upon request via this website (http://yhs.cdc.go.kr).

### Measurements

#### BMI, perceived weight status, and estimation of weight status

Self-reported body weight and height were used to calculate body mass index (BMI, weight in kilograms divided by square of height in meters). Weight status was classified into overweight/obese (BMI? *a*?85th percentile), normal (5th≤BMI<85th percentile), and underweight (BMI<5th percentile) based on sex- and age-specific reference according to the 2007 Korean National Growth Chart [45–47]. Perceived weight status was measured by the question, “How would you describe your weight,” with response options of “very underweight,” “slightly underweight,” “about the right weight,” “slightly overweight,” and “very overweight.” The answers were reclassified into three categories, overweight/obese (very overweight or slightly overweight), normal (about the right weight), and underweight (slightly underweight or very underweight) status [48].The outcome of interest, weight status estimation, was classified into three categories comparing self-reported versus perceived weight status: (a) overestimation (perceived weight status > objective weight status); (b) accurate estimation (perceived weight status = objective weight status); and (c) underestimation (perceived weight status < objective weight status).

#### Demographic variables

Demographic characteristics included age, sex, family income status, parental education levels, family structure, year in school, school type by gender composition (single sex or co-education schools), and academic achievement. Family income was assessed by the question, “What is your family economic status?” The five possible response categories ranging from very low to very high were clumped into three categories: high (very high or high), middle (middle), and low (low or very low) [44]. Paternal and maternal education levels were assessed, respectively, with the following options: “less than or equal to middle school graduate,” “high school graduate,” “more than or equal to college graduate,” “don’t know,” and “not applicable (in case an individual’s father/mother was not alive).” We reclassified the measures into four categories: “(1) less than or equal to high school graduate,” “(2) more than or equal to college graduate,” “(3) don’t know,” and “(4) not applicable.” Academic performance was evaluated by asking, “During the past 12 months, how would you rate your academic performance?” with options of “high,” “middle-high,” “middle,” “middle-low,” and “low.” The response was reclassified into three categories, high (high or middle-high), middle (middle), and low (middle-low or low) [44].

#### School and neighborhood environments

School- and neighborhood-level covariates included school type (middle, high, and vocational high), sex composition (single-sex, and coeducational schooling), and school average BMI, geographic region(big, mid/small city, and rural areas). Average school BMI was calculated to assess school norms regarding the ideal body weight by aggregating the self-reported BMI of individuals in each cluster. Prior studies have reported that BMI of significant others including parents, friends, and classmates can influence an individual’s weight status perception and weight change [28, 49–52]. Between two commonly used approaches of measuring ecological exposure by aggregating individual responses, we chose to use the *self-included measure*, where average school BMI for i^th^ individual at j^th^ school is calculated by utilizing BMI of every individual at j^th^ school including the i^th^ individual, rather than the *self-excluded measure*, where average school BMI for i^th^ individual at j^th^ school is calculated based on BMI of all the other individuals at j^th^ school except for that of the i^th^ individual, as the potential implication and intervention for school norms would be focused at school level rather than individual level [53, 54].

#### Behavioral characteristics

Health-related behavioral characteristics included physical activity, weight control behaviors, illicit substance use including alcohol, tobacco, and drugs, and sexual experience. Physical activity was assessed by asking, “During the past 7 days, on how many days were you physically active (any kind that increased your heart rate and made you breathe hard) for a total of at least 60 minutes per day?” The eight possible responses from 0 to 7 days per week were reclassified into three categories, desirable (? *a*?3days/week), low amount (1–2 days/week), and no weekly physical activity [44, 55]. Tobacco smoking and alcohol drinking were measured by asking lifetime experiences with the following questions, “Have you ever tried cigarette smoking, even one or two puffs?” and “Have you ever had at least one drink of alcohol except during ancestral rites or religious ceremonies?” [44, 55] Sexual behavior was assessed by asking whether an individual (a) ever had sexual intercourse with either same- or different-sex partner(s), or (b) never had. [44]. Illicit drug use was evaluated by asking, “Have you ever inhaled/taken substances such as glue, butane gas, stimulant, marijuana, amphetamine, heroin, high-dose cold medicine, or anxiolytics for the purpose of mood elevation, hallucination, or excessive dieting?” with three response options, “No,” “Had ever used in the past but do not currently,” and “Use currently” A new variable, drug experience, was categorized into (a) never and (b) ever experienced [56].

### Statistical analysis

First, we examined the proportion of each weight status misperception pattern (WSMP) among Korean adolescents, baseline characteristics of the study population, and the distribution of each WSMP within each level of potential predictors by performing ANOVA test for continuous predictors, and chi-squared test for binary/categorical predictors. Then, we performed sex-stratified multilevel random intercept multinomial logistic regression models, where students (level 1) were nested within schools (level 2), in order to investigate the associations between each WSMP and factors at individual-, family-, and school-level, simultaneously [57]. Covariates included age, individual BMI, academic achievement, physical activity, sexual experience, substance uses (tobacco, alcohol, illicit drug), household income, parental education, family structure, geographic region, school type (middle/high/vocational high), school sex composition, and school average BMI. We also examined whether there was significant residual between schools variation in the distribution of each WSMP after adjusting for all predictors at fixed part.

For preliminary investigation from creating variables to performing ANOVA and chi-squared tests for Tables 1–4, we used SAS 9.4 (SAS Institute, Inc. Cary, NC). For all multilevel analyses, we used MLwiN 2.28 (Center for Multilevel Modeling, Bristol University, London, UK) with the 2nd order penalized quasi-likelihood (PQL) method for unordered multinomial distributional estimation according to the User’s Manual (Center for Multilevel Modeling, Bristol University, London, UK) [58]. For all statistics and analyses, sampling weight was not applied as the focus of our investigation was to investigate the association between WSMP and factors at multiple levels, rather than estimating nationally representative parameters [40]. Statistical significance was set as p-value of 0.05 (two-sided) for testing fixed terms, and p-value of 0.05 (one-side) for testing residual variation in the random intercept at level 2 based on prior literature [40].

**Table 1:** Cross Tabulation of Self-reported and Perceived Body Weight Status among Korean Adolescents aged 12–18 years old^a^,^b^.

		Self-perceived weight			
BMI categories^c^		Underweight (%)	Normal (%)	Overweight (%)	Total (%)
**All (N = 72,228)**					
	Underweight	91.1	7.0	1.9	6.0
	Normal	28.9	40.9	30.2	80.7
	Overweight/obese	0.2	3.1	96.7	13.3
	Total	28.8	33.9	37.3	
**Girls (N = 34,999)**					
	Underweight	87.5	10.0	2.5	5.9
	Normal	19.7	43.1	37.2	82.1
	Overweight/obese	0.1	2.5	97.4	12.0
	Total	21.4	36.2	42.4	
**Boys (N = 37,229)**					
	Underweight	94.3	4.5	1.2	6.1
	Normal	37.7	38.8	23.5	79.8
	Overweight/obese	0.3	3.4	96.3	14.1
	Total	35.8	31.8	32.4	

Abbreviations: BMI, body mass index.

a) Source data: the Eighth Korea Youth Risk Behavior Survey (KYRBS, 2012)

b) Proportions were calculated by using raw frequencies among the study population (N = 72,228) without adjusting for sampling weights

c) BMI was classified into three categories based on age- and sex-specific BMI reference from the 2007 Korea national growth chart: underweight (BMI<5th percentile), normal weight (5th≤BMI<85th), and overweight/obese (BMI≥85th percentile).

**Table 2:** Descriptive Statistics among Girls and Boys aged 12–18 in the Study Population (N = 72,228)^a^.

	Overall (N = 72,228)	Girls (N = 34,999)	Boys (N = 37,229)
Weight Status Estimation (%)			
Accurate	51.4	52.5	50.3
Underestimate	23.7	16.4	30.6
Overestimate	24.9	31.1	19.1
Individual level			
Age: years (SD)	14.9 (1.8)	14.9 (1.8)	14.9 (1.8)
BMI: kg/m2 (SD)	20.6 (3.0)	20.3 (2.7)	20.9 (3.3)
Academic Achievement (%)			
High	34.6	33.7	35.5
Middle	26.9	27.7	26.2
Low	38.5	38.6	38.4
Physical Activity (%)			
> = 3days/week	30.4	19.5	40.5
1-2days/week	32.6	33.6	31.8
none/week	37	46.9	27.7
Tobacco Use (%)			
Ever	24.4	15.6	32.7
Alcohol Use (%)			
Ever	47	42.9	50.9
Drug Use (%)			
Ever	1.0	0.8	1.2
Sexual Experience (%)			
Ever	4.0	2.3	5.5
Family level			
Household Income (%)			
High	30	27	32.9
Middle	47.3	49.5	45.2
Low	22.7	23.5	21.9
Maternal Education (%)			
< = high school graduate	48.4	50.9	46
> = college graduate	34.7	34.5	34.8
Unknown	13.3	11.4	15.1
N/A	3.7	3.2	4.1
Paternal Education (%)			
< = high school graduate	38.7	39.6	37.7
> = college graduate	43.8	43.6	44
Unknown	13.7	12.8	14.5
N/A	3.9	4.0	3.8
Family structure (%)			
living w/ both parents	83.1	83.1	83.2
School & Neighborhood levels			
School type (%)			
middle	50.4	50.6	50.1
general high	38.4	37.8	39
vocational high	11.2	11.6	10.9
Sex-composition (%)			
Single-sex	33.2	34.3	32.2
School Average BMII: kg/m2 (SD)	20.6 (0.7)	20.5 (0.6)	20.7 (0.8)
Living placement (%)			
Big city	45.3	44.4	46.3
Mid/Small city	43.4	44.6	42.2
Rural area	11.3	11	11.5

a) Source Data: the Eighth Korea Youth Risk Behavior Web-based Survey, 2012.

**Table 3:** Sex-Stratified Distribution of Each Weight Status Estimation Pattern Across Levels of Individual, Family, and School-Level Characteristics Among the Study Population (N = 72,228)^a,b^.

		GIRLS (N = 34,999)				BOYS (N = 37,229)		
	Accurate	Under	Over		Accurate	Under	Over	
Individual level								
Age (years)	14.9	14.5	15.1	< .0001	14.9	14.9	14.8	< .0001
BMI (kg/m2)	20.5	18	21.2	< .0001	21.7	18.7	22.2	< .0001
Academic Achievement (%)								
High	52.1	18.3	29.6	< .0001	49	31.8	19.2	0.0005
Middle	53.9	15.4	30.7		51.6	29.3	19.2	
Low	51.8	15.6	32.6		50.7	30.4	18.9	
Physical Activity (%)								
> = 3days/week	53	16.5	30.5	0.56	50.8	31.3	17.9	< .0001
1-2days/week	52.4	16.7	30.9		49.5	30.1	20.4	
none/week	52.4	16.2	31.4		50.5	30.1	19.4	
Tobacco Use (%)								
Ever	50.1	14.8	35.2	< .0001	49.5	32.7	17.9	< .0001
Never	52.9	16.8	30.3		50.7	29.6	19.7	
Alcohol Use (%)								
Ever	51	14.8	34.2	< .0001	50.2	31.1	18.7	0.04
Never	53.6	17.7	28.7		50.5	30.1	19.5	
Drug Use (%)								
Ever	53.1	18.3	28.6	0.55	50.4	30.6	19.1	0.32
Never	52.5	16.4	31.1		46.8	32.4	20.8	
Sexual Experience (%)								
Ever	49.6	17.8	32.6	0.25	48.6	34.6	16.7	< .0001
Never	52.5	16.4	31.1		50.4	30.4	19.2	
Family level								
Household Income (%)								
High	53.6	18.8	27.6	< .0001	50.9	29.8	19.4	0.014
Middle	53.5	15.7	30.8		50.6	30.7	18.8	
Low	49.1	15.2	35.7		48.9	31.7	19.4	
Maternal Education (%)								
< = high school graduate	52.3	15.5	32.1	< .0001	51.1	30.5	18.4	0.0001
> = college graduate	52.1	17.6	30.3		49	31	20	
Unknown	54.2	17.1	28.7		51.6	29.2	19.3	
N/A	53	16	31.1		48.4	33.1	18.6	
Paternal Education (%)								
< = high school graduate	52.6	15.3	32.1	< .0001	51	30.5	18.5	< .0001
> = college graduate	52.1	17.4	30.5		49.2	31.4	19.4	
Unknown	53.5	17	29.5		52.3	27.9	19.9	
N/A	51.8	15.6	32.6		48.7	32.9	18.4	
living w/ both parents (%)								
Yes	52.7	16.5	30.8	0.02	50.6	30.3	19.1	0.03
No	51.1	16.4	32.6		49	31.9	19.1	
School & Neighborhood levels								
School type (%)								
middle	53.1	19.2	27.7	< .0001	50.2	29.9	19.9	0.0001
general high	51.8	13.9	34.4		50.1	31.3	18.6	
vocational high	52	12.9	35		51.3	31.5	17.2	
Sex-composition (%)								
Single-sex schooling	52.7	15.7	31.6	0.01	50.6	29.8	19.7	0.03
Co-educational	52.3	16.9	30.8		50.2	31	18.8	
School Average BMI (kg/m2)	20.47	20.37	20.51	< .0001	20.74	20.71	20.71	< .0001
Living placement (%)								
Big city	52.6	16.4	31	0.9	50.1	30.5	19.4	0.5
Mid/Small city	52.2	16.5	31.3		50.6	30.5	19	
Rural area	52.9	16.5	30.6		50.1	31.5	18.5	

a) Source Data: the Eighth Korea Youth Risk Behavior Web-based Survey, 2012.

b) For all binary/categorical predictors, row percentage was presented.

**Table 4:** Sex-stratified Multilevel Random Intercept Multinomial Logistic Regression Models for Weight Status Misperception Patterns Among Korean Adolescents Aged 12–18 (N = 72,228)^a^.

	GIRLS (N = 34,999)						BOYS (N = 37,229)					
	Underestimation^b^^,^^c^			Overestimation^b^^,^^d^			Underestimation^b^^,^^c^			Overestimation^b^^,^^d^		
Fixed Part	OR	95%	CI	OR	95%	CI	OR	95%	CI	OR	95%	CI
Individual level												
age (years)	0.95*	0.92	0.98	1.05*	1.02	1.08	1.08*	1.05	1.11	0.97	0.95	1.001
BMI (kg/m2)	0.62*	0.61	0.63	1.11*	1.10	1.13	0.67*	0.66	0.68	1.06*	1.06	1.07
academic achievement (ref: low)												
high	1.10*	1.01	1.18	1.00	0.94	1.06	1.13*	1.06	1.20	1.05	0.98	1.12
middle	0.91*	0.84	0.99	0.96	0.90	1.02	0.97	0.91	1.03	1.00	0.94	1.08
physical activity (ref: none/week)												
1-2days/week	1.05	0.98	1.13	0.99	0.94	1.04	1.04	0.98	1.11	1.06	0.99	1.13
> = 3days/week	1.06	0.97	1.15	0.98	0.92	1.04	1.12*	1.05	1.19	0.89*	0.83	0.95
sexual experience (ref: none)												
ever experienced	1.22	0.99	1.51	0.94	0.80	1.10	1.15*	1.03	1.28	0.97	0.85	1.10
substance use (ref: never)												
tobacco (ever used)	1.01	0.92	1.11	1.11*	1.04	1.19	1.11*	1.05	1.18	0.96	0.90	1.03
alcohol (ever used)	1.06	0.98	1.13	1.11*	1.06	1.17	1.04	0.99	1.11	1.02	0.96	1.08
illicit drug (ever used)	1.02	0.73	1.41	0.90	0.69	1.17	1.04	0.83	1.31	1.21	0.95	1.55
family level												
household income (ref: low)												
high	0.92	0.83	1.01	0.76*	0.71	0.82	0.89*	0.83	0.96	0.89*	0.82	0.97
middle	0.83*	0.77	0.90	0.84*	0.80	0.89	0.91*	0.85	0.97	0.92*	0.86	0.98
paternal education (ref: < = high school graduate)												
> = college graduate	1.03	0.95	1.12	1.03	0.97	1.10	1.06	0.997	1.14	1.02	0.95	1.10
unknown	0.97	0.85	1.11	1.04	0.94	1.15	0.84*	0.76	0.93	1.02	0.92	1.14
N/A	0.89	0.75	1.07	0.99	0.87	1.13	1.04	0.91	1.20	0.97	0.83	1.13
maternal education level (ref: < = high school graduate)												
> = college graduate	1.00	0.92	1.08	1.02	0.96	1.09	1.03	0.97	1.10	1.11*	1.04	1.19
unknown	0.96	0.84	1.09	0.92	0.83	1.02	1.03	0.94	1.14	1.01	0.90	1.12
N/A	0.90	0.75	1.10	0.93	0.80	1.07	1.03	0.90	1.18	1.05	0.90	1.22
living with both parents (yes vs no)	0.89*	0.81	0.98	1.00	0.93	1.07	0.97	0.90	1.05	0.95	0.88	1.04
school & neighborhood levels												
regional area (ref: rural)												
big city	0.90	0.80	1.01	1.01	0.93	1.10	0.94	0.86	1.03	1.02	0.93	1.12
mid/small city	0.96	0.85	1.07	1.03	0.95	1.12	0.93	0.85	1.02	1.00	0.91	1.10
school type (ref: middle school)												
general high school	1.03	0.89	1.19	1.10	0.99	1.23	1.12	0.995	1.26	1.01	0.90	1.15
vocational high school	0.96	0.80	1.15	1.07	0.95	1.22	1.01	0.88	1.17	0.95	0.82	1.10
school sex composition (ref: coeducational)												
single-sex school	1.13*	1.04	1.22	0.95	0.90	1.005	0.96	0.89	1.03	1.07	0.99	1.15
school average BMI (kg/m2)	1.19*	1.09	1.30	0.86*	0.81	0.92	1.15*	1.08	1.23	0.92*	0.86	0.98
Random Part	residual variance	SE		residual variance	SE		residual variance	SE		residual variance	SE	
random intercept	0.04**	0.01		0.01	0.01		0.03**	0.01		0.01	0.01	

Abbreviations: BMI, body mass index; OR, odds ratio; CI, confidence interval; N/A, non-applicable response.

a) Source data: the Eighth Korea Youth Risk Behavior Web-based Survey, 2012.

b) The reference group was set as adolescents with accurate weight status estimation.

c) ORs refer to the odds of weight status underestimation (WSUE), compared to accurate estimation.

d) ORs refer to the odds of weight status overestimation (WSOE), compared to accurate estimation.

e) *: two-sided P<0.05.

f) **: one-sided P<0.05.

## Results

### The Proportions of Weight Status Under- and Overestimation

The descriptive statistics and proportions of each weight status misperception pattern (WSMP) among Korean adolescents aged 12–18 from KYRBS 2012 were presented in Tables 1 & 2.

As presented in Table 1, among the Korean adolescents aged 12–18 years, 80.8% (82.1% among girls, 79.8% among girls) had normal weight status based on self-reported weight and height. However, of these, more than half (56.9% among girls, 61.2% among boys) misperceived their weight status. Table 2 displayed the overall proportion of weight status underestimation (WSUE) as 23.7% (16.4% among girls, 30.6% among boys), and overestimation (WSOE) as 24.9% (31.1% among girls, 19.1% among boys). Among adolescents with being overweight/obese weight status (12.0% among girls, 14.1% among boys), 2.5% of girls, and 3.7% of boys underestimated their weight status. Among those with underweight status (5.9% among girls, 6.1% among boys), 12.5% of girls, and 5.7% of boys overestimated their weight status.

### The Sex-stratified Distribution of Weight Status Underestimation and Overestimation across Predictors

Table 3 presented the sex-stratified distributions of weight status underestimation (WSUE) and overestimation (WSOE) across each level of factors at individual-, family-, and school-level.

In terms of school contexts, single-sex schooling, compared with coeducational schooling, had significantly different distribution of weight status misperception patterns (WSMPs) among both girls (p<0.01) and boys (p<0.03). Among girls attending single-sex schools, the proportions of weight status overestimation (WSOE) and underestimation (WSUE) were 31.6% and 15.7%, respectively. However, among girls attending coeducational schools, the proportions of WSOE and WSUE were 30.8% and 16.9%, respectively (The p-value for the global chi-squared test with 2 degrees of freedom was smaller than 0.01). Similar pattern was observed among boys as well. Among boys attending single-sex schools, the proportions of WSOE and WSUE were 19.7% and 29.8%, while the proportions among boys attending coeducational schools were 18.8% for WSOE, and 31.0% for WSUE.

At the family level, there was significantly different distribution of weight status misperception patterns (WSMPs) across household income categories (the p-value for the global test with 4 degrees of freedom: < .0001 for girls, and 0.014 for boys). Among girls, the proportions of WSOE were 35.7% among those living with low household income, 30.8% among middle, and 27.6% among high. Among boys, the proportions of WSUE were 31.7% among those living with low household income, 30.7% among middle, and 29.8% among high.

At the individual level, significantly different distributions of WSMPs were observed across the levels of academic achievement (the p-value for the global test with 4 degrees of freedom: < .0001 for girls, and 0.0005 for boys.) Among girls, the proportions of WSOE were 32.6% among those with low academic achievement, 30.7% among middle, and 29.6% among high. Among boys, the proportions of WSUE were 31.8% among those with high academic achievement, 29.3% among middle, and 30.4% among low.

### Sex-stratified Multilevel Random Intercept Multinomial Logistic Models

The results from the sex-stratified multilevel multinomial logistic models with random intercept were presented in Table 4. In terms of school and neighborhood environments, after adjusting for all other covariates, school average BMI and school sex composition predicted both WSUE and WSOE for boys and girls. One unit higher school average BMI (kg/m^2^) was associated with greater probability of WSUE (OR = 1.19, 95% CI: 1.09, 1.29 for girls, OR = 1.15, 95% CI: 1.08, 1.23 for boys), and smaller probability of WSOE (OR = 0.86, 95% CI: 0.81, 0.92 for girls, OR = 0.92, 95% CI: 0.86, 0.98 for boys). In addition, among girls, single-sex schooling, when compared with coeducational schooling, was associated with greater odds of WSUE (OR = 1.13, 95% CI: 1.13, 1.22). Though not statistically significant, the directionality was the opposite for WSOE comparing single-sex versus coeducational schooling among girls (OR = 0.95, 95% CI: 0.90, 1.01). Interestingly, though not statistically significant, the opposite pattern was observed among boys. When comparing single-sex versus coeducational schooling, the directionality of the point estimate for WSUE was negative (OR = 0.96, 95% CI: 0.86, 1.03), and for WSOE was positive (OR = 1.07, 95% CI: 0.99, 1.15). Of note, there was significant between schools variation in the distribution of WSUE among boys (residual variance in logit scale = 0.04 with SE = 0.01) and girls (residual variance in logit scale = 0.03 with SE = 0.01), after adjusting for all fixed terms. However, we failed to find significant between schools variation for WSOE among boys (residual variance in logit scale = 0.01 with SE = 0.01) and girls (residual variance in logit scale = 0.01 with SE = 0.01) at one-side p-value of 0.05.

In terms of family environments, household income, parental education, and family structure were significantly associated with WSMPs among Korean adolescents. Higher household income (high vs low, middle vs low) was mostly negatively associated with both WSUE and WSOE. Among boys, both high and middle levels of household income, when compared with low, had negative associations with WSUE (OR for high vs low = 0.89, 95% CI: 0.83, 0.96, OR for middle vs low = 0.91, 95% CI: 0.85, 0.97) and WSOE (OR for high vs low = 0.89, 95% CI: 0.82, 0.97, OR for middle vs low = 0.92, 95% CI: 0.86, 0.98). Similarly, among girls, high and middle levels of household income, when compared with low, had negative associations with WSOE (for high vs low, OR = 0.76, 95% CI: 0.71, 0.82, and for middle vs low, OR = 0.84, 95% CI: 0.80, 0.90) and WSUE (for middle vs low, OR = 0.83, 95% CI: 0.77, 0.90), except for WSUE comparing high versus low household income levels among girls (OR = 0.92, 95% CI: 0.84, 1.02). Maternal education equal to or more than college graduate, when compared with equal to or less than high school graduate, was associated with greater probability of WSOE among boys (OR = 1.11, 95% CI: 1.04, 1.19). Living with both parents, when compared to living with one or none of the adolescent’s parents, was associated with smaller probability of WSUE among girls (OR = 0.89, 95% CI: 0.81, 0.98).

At the individual level, age, BMI, academic achievement, physical activity, substance uses including tobacco and alcohol, and sexual experience were significantly associated with WSMPs.

Individuals responded to have “high/middle-high” level of academic achievement, when compared to “low/middle-low,” were more likely to have weight status underestimation (WSUE) among girls (OR = 1.09, 95% CI: 1.01, 1.18) and boys (OR = 1.13, 95% CI: 1.06, 1.20). Interestingly, individuals responded to have “middle/middle-low” level of academic achievement, when compared to “low/middle-low,” were less likely to have WSUE among girls (OR = 0.92, 95% CI: 0.85, 0.995), while the association was not significant among boys (OR = 0.97, 95% CI: 0.91, 1.03). Having regular physical activity at least three days per week with of at least 60 minutes per each day, compared with none, was positively associated with WSUE (OR = 1.11, 95% CI: 1.05, 1.18) and negatively associated with WSOE (OR = 0.89, 95% CI: 0.84, 0.96) among boys. While the associations were not statistically significant, the pattern of the associations in terms of the directionality was the same among girls (for WSUE, OR = 1.06, 95% CI: 0.97, 1.15, and for WSOE, OR = 0.97, 95% CI: 0.91, 1.04).

Lifetime tobacco use was positively associated with WSOE among girls (OR: 1.10, 95% CI: 1.03, 1.18), and with WSUE among boys (OR: 1.10, 95% CI: 1.04, 1.17).Lifetime alcohol use was positively associated with WSOE among girls (OR: 1.12, 95% CI: 1.06, 1.18). Though it was not statistically significant, the directionality of the association between lifetime alcohol use and WSUE among boys was positive (OR: 1.05, 95% CI: 0.99, 1.11). Lastly, having sexual experience was positively associated with WSUE among both girls (OR: 1.25, 95% CI: 1.02, 1.54) and boys (OR: 1.17, 95% CI: 1.05, 1.30).

## Discussion and Conclusions

In this present study, we investigated risk and protective factors for WSMPs at individual-, family- and school-level, stratified by sex, whether there was significant residual between school variations in the distribution of WSUE and WSOE among Korean adolescents aged 12–18 years. The current study showed four key findings as followings. First, school environmental factors such as school average BMI and sex composition predicted adolescents’ weight status misperception patterns (WSMPs). Among both boys and girls, individuals attending a school with greater average BMI were more likely to underestimate, and less likely to overestimate their body weight status. Among girls, single-sex schooling was positively associated with weight status underestimation (WSUE). Second, family contextual factors such as household income, maternal education, and family structure were associated with WSMPs. Among both boys and girls, lower household income was associated with higher probability of any type of weight status misperception (both WSUE and WSOE). Among boys, higher maternal education (equal to or more than college graduate versus equal to or less than high school graduate) was positively associated with WSOE. Among girls, living with both parents was inversely associated with weight status underestimation (WSUE). Third, at individual level, age, BMI, academic achievement, physical activity, tobacco and alcohol use, and sexual experience predicted WSMPs. Among both boys and girls, high academic achievement (high/middle-high), compared with low (low/middle-low), was positively associated with WSOE. Among boys, at least 3 days of physical activity (> = 1 hour per day) over the past week, compared with no physical activity (> = 1 hour per day) over the past week, was positively associated with WSUE, and inversely associated with WSOE. Lifetime tobacco and alcohol use were positively associated with WSOE among girls. Sexual experience was positively associated with WSUE among boys. Lastly, after adjusting for all fixed terms listed above, there was significant residual between schools variation in the distribution of WSUE among boys and girls, but not WSOE.

In terms of the potential role of school environments in shaping adolescents’ weight misperception patterns, a previous study by Maximova et al. reported that overweight/obese individuals with greater school average BMI were more likely to underestimate their weight status than their counterparts, after adjusting for age, sex, parent BMI, and socioeconomic status at family (household income, parental education) and school (percentage of mothers without high school education in a given school) levels, among overweight and obese Canadian adolescents aged 9, 13, and 16 years [28]. Building on previous studies, we investigated whether school average BMI was associated with weight status overestimation as well as underestimation among Korean adolescents. As we expected, the results from the present study showed that girls and boys with greater school average BMI were more likely to underestimate their weight status among Korean adolescents aged 12–18 years, consistent with the previous research among Canadian adolescents. Additionally, individuals with greater school average BMI were less likely to overestimate their weight status. We attribute this to the influence of school norms on weight perception.

As another school level aspect, we also tested whether school sex-composition was associated with students’ weight status misperception patterns. A recent study by Choi et al. presented that, among high school students (10th-12th graders) in Seoul, South Korea, individuals attending single-sex schools, compared with those attending coeducational schools, have, on average, greater BMI (0.20 kg/m^2^ for girls, 0.17 kg/m^2^ for boys) after adjusting for age, parental education, family structure, and household income [37]. As a potential mechanisms linking school sex composition and weight gain, the authors suggested that sex composition of a school could influence perception of ideal body shape among students [37]. The findings from our current study showed that female adolescents aged 12–18 years attending single-sex schools, when compared with their counterparts attending coeducational schools, were more likely to underestimate their weight status, after adjusting for factors at individual, family, and school levels. Though we failed to find significant association between school sex composition and weight status overestimation among girls, and any weight status misperception patters among boys, the findings from the present study suggested that single-sex schooling, compared to coeducational schooling, could contribute to shaping less stringent norm regarding ideal body shape in terms of thinness among female adolescents.

In terms of between schools variation in weight status misperception, Maximova et al. investigated whether there was significant between schools variation in weight status misperception among Canadian adolescents aged 9, 13, and 16 years [28]. After creating the misperception score, calculated by the difference between perceived weight Z-score (based on the Stunkard Body Rating Scale) and BMI Z-score, the authors performed a multilevel linear regression model where students (level 1) were nested to schools (level 2). The estimated residual variances for random intercept term (level 2) were significant at alpha of 0.05 across all age groups (9, 13, and 16 years) [28]. Building on the authors investigations, we performed sex-stratified multilevel random intercept multinomial logistic model where students (level 1) were nested to schools (level 2), specifically asking whether there was significant residual between-schools variation for each weight status misperception type (WSUE and WSOE, separately) at one-sided alpha of 0.05, after adjusting for all individual, family, and school-level factors at fixed part. One of the unique features of our approach was that it could provide evidence regarding between schools variation for WSUE and WSOE, respectively. The results from the current analysis suggested that, after adjusting for all factors at fixed part, there was significant residual between schools variation for WSUE among girls and boys, while significant residual between schools variation for WSOE was not detected. While further studies are necessary to clarify the association between school environments and adolescents weight status estimation, the findings from the current study suggested that school environments played significant roles in shaping adolescents’ weight status misperception patterns.

At family level, household income, paternal education, and family structure predicted adolescents’ weight status misperception patterns. There were mixed findings with respect to the association between weight status misperception and SES. While inverse association between household income and weight status misperception among adolescents has been more commonly reported [29, 30, 33, 59], some other studies reported either no significant association between household income and weight status misperception [60, 61], or positive association between SES (measured by incorporating both household income and parental education) and weight status overestimation among girls [4]. The findings from the present study suggested that higher household income background was associated with lower probability of weight status over- and underestimation. Additionally, living with both parents, when compared to living with either one or none of parents, was inversely associated with WSUE among girls. However, contrastingly, higher maternal education (> = college graduate versus < = high school graduate) was associated with greater probability of WSOE among boys. A conflicting finding from a previous study by Xie et al. was reported among Chinese middle and high school students that higher parental education was inversely associated with WSOE among girls [62].

At the individual level, age, BMI, academic achievement, physical activity, sexual experience, tobacco and alcohol use were significantly associated with weight status misperception among Korean adolescents. In terms of academic achievement, a previous study by Fan et al. reported that a higher average GPA (each average GPA category of A-D compared with F) was positively associated with WSUE among overweight/obese US adolescents, and inversely associated with WSOE among non-overweight/obese US adolescents except for Hispanic female adolescents [13]. Similarly, in our present study, Korean high academic achievement, compared with low academic achievement, was positively associated with WSUE for both girls (OR = 1.10, 95% CI: 1.01, 1.18) and boys (OR = 1.13, 95% CI: 1.06, 1.20). However, we failed to find significant association between academic achievement and WSOE. Additionally, the directionality of the association became the opposite for WSUE comparing middle versus low academic achievement among girls (OR = 0.91, 95% CI: 0.84, 0.99). Fan et al. also reported that having sexual experience was inversely associated with WSOE among non-overweight/obese US female and male adolescents, and positively associated with WSUE among overweight/obese female and male adolescents, except for white female adolescents [13]. Similarly, in our present study, sexual experience was positively associated with WSUE among Korean male adolescents (OR = 1.15, 95% CI: 1.03, 1.28). Though not statistically significant, the same directionality of the point estimate was observed for WSUE among Korean female adolescents (OR = 1.22, 95% CI: 0.99, 1.51).

The present study had several limitations. First, due to the cross-sectional nature of the national survey, drawing a causal inference based on the findings from this present study may not be valid. Longitudinal studies are necessary to further investigate risk and protective factors for weight status misperception. Second, BMI was calculated by self-reported weight and height, leading to potential bias due to measurement error. Previous studies reported that girls were more likely to under-report their weight, while boys were more likely to over-report their height, resulting in underestimating true BMI [63–65].However, a validation study for the 4^th^ KYRBS (2008) revealed that that the self-reported BMI and measured BMI had good/acceptable degree of agreement with the kappa statistics of 0.79 (95% CI: 0.70, 0.88). The sensitivity and specificity of the self-reported measure to assess obesity were 69.0% and 100%, respectively [66]. Studies from other populations also reported that self-reported weight and height provided relatively accurate measure, and can be used as valid tool to estimate BMI when measured weight and height are not available [66, 67]. Additionally, when measuring perceived weight status, we collapsed five response categories (“very thin” to “very overweight”) into three (“very/slightly thin,” “about the right,” and “slightly/very overweight”), which was compared to three categories of actual weight status, based on self-reported weight and height, “underweight” (<5th percentile), “normal” (5th< = BMI<85th)”, and “overweight/obese” (85th< = BMI percentile). Therefore, there was only one option for individuals with normal weight status to be evaluated as “accurate weight status estimation” while there were two options in the original measure for individuals with underweight and overweight/obese status, respectively, inducing more possibility of inaccuracy of weight status estimation [28]. Therefore, further studies with not only objectively measured weight and height, but also more precise measurement of perceived weight status are needed to provide valid evidence of risk factors for weight status misperception among adolescents [28].However, the strength of the present study included the use of multilevel multinomial logistic modeling approach based on a nationally representative sample of Korean adolescents, which allowed us to investigate the risk and protective factors for both weight status underestimation (OWUE) and overestimation (OWOE) among Korean adolescents at individual-, family-, and school-level, simultaneously, and residual between schools variations in the distribution of OWUE and OWOE, respectively. Additionally, to our knowledge, this is the first study to investigate the association between school sex composition and weight status misperception.

In line with the growing body of literature suggesting weight status misperception among adolescents as a significant public health issue [1, 4, 12, 13, 21, 22, 24, 32, 59, 68–70], the findings from this study can provide evidence regarding risk factors for weight status underestimation and overestimation among adolescents. In particular, building on previous studies investigating the influence of social contexts on adolescents’ body weight, weight status perception, and weight control behaviors [35, 36, 38, 39], the present study suggest that school environmental factors such as school average BMI and sex composition, as well as family contexts such as household income, parental education, and family structure need to be considered when developing and implementing obesity prevention interventions. More importantly, further studies utilizing prospective designs with objectively measured anthropometric information are needed to provide more valid evidence with respect to risk and protective factors for weight status misperception among adolescents.
